# Effects of palladium on the optical and hydrogen sensing characteristics of Pd-doped ZnO nanoparticles

**DOI:** 10.3762/bjnano.5.140

**Published:** 2014-08-13

**Authors:** Anh-Thu Thi Do, Hong Thai Giang, Thu Thi Do, Ngan Quang Pham, Giang Truong Ho

**Affiliations:** 1 Institute of Materials Science, Vietnam Academy of Science and Technology, 18 Hoang Quoc Viet, Caugiay, Hanoi, Vietnam; Tel.: +84-43-7569318; Fax: +84-43-8360705

**Keywords:** carrier dynamics, hydrogen sensing, Pd-doped ZnO, photoluminescence, sensor

## Abstract

The effect of palladium doping of zinc oxide nanoparticles on the photoluminescence (PL) properties and hydrogen sensing characteristics of gas sensors is investigated. The PL intensity shows that the carrier dynamics coincides with the buildup of the Pd-related green emission. The comparison between the deep level emission and the gas sensing response characteristics allows us to suggest that the dissociation of hydrogen takes place at Pd_Zn_-vacancies ([Pd ^2+^(4d^9^)]). The design of this sensor allows for a continuous monitoring in the range of 0–100% LEL H_2_ concentration with high sensitivity and selectivity.

## Introduction

Semiconductor zinc oxides (ZnO) nanocrystals are not only interesting for fundamental physics, but they are also important for both optoelectronic and emerging electronic device applications, in particular for hydrogen sensing [1–6]. The key features and availability of ZnO nanocrystals in distributed discrete gas sensing devices crucially depend on the growth conditions. These conditions strongly influence their size, uniformity and defects. Optical properties and gas sensing characteristics in ZnO nanostructures are mainly expected to differ in terms of their quality from those in bulk materials. In ZnO bulk material, the sensitivity and selectivity are not sufficiently high. ZnO nanocrystals possess a large surface atom/bulk atom ratio [7], which corresponds to a higher sensitivity, thermal stability [8], compatibility with other nanodevices, and are potentially the best gas sensors. Oxides cannot easily distinguish between different types of gases, but the addition of certain noble metals as dopants can promote the gas-sensing performance [9–11]. Noble metal dopants in ZnO can modify the optical and electric properties of ZnO, which influence the sensitive performance [10,12]. Palladium, a 4d metal, is taken as an impurity in ZnO. This is most likely caused by their special electronic configuration, i.e., 4d. Due to the sensitivity of the palladium ions with deep holes on singly ionized oxygen interstitials, Zn anti-site vacancies, and oxygen vacancies, it is of interest to find out whether Pd incorporated in ZnO significantly improves sensitivity and specificity for hydrogen [13–14].

In this work, we have successfully synthesized Pd-doped ZnO nanoparticles for an application as gas sensors by a low-temperature wet-chemical process. Photoluminescence (PL) measurements at room temperature are then carried out in order to determine the role of vacancies, trapping levels, and the transition shift of the PL emission maximum in these samples. In order to study and optimize the four main factors affecting the ability of hydrogen sensors, typical sensors based on ZnO nanoparticles have been designed. The hydrogen gas sensor based on Pd-doped ZnO shows a relative fast response compared with the undoped sample. We also investigated the hydrogen sensing characteristics of these catalytic gas sensors in the measurement chamber containing hydrogen in air, with concentrations of 25–100% of the lower explosive limit (LEL), which is the minimum concentration of vapor or gas in air below which flame propagation does not occur on contact with a source of ignition [15]. The value of 25–100% LEL is equivalent to about 10,000–40,000 ppm. An existing correlation between the PL emissions and hydrogen sensing characteristics of these gas sensors will also be discussed.

## Results and Discussion

**Figure 1:**
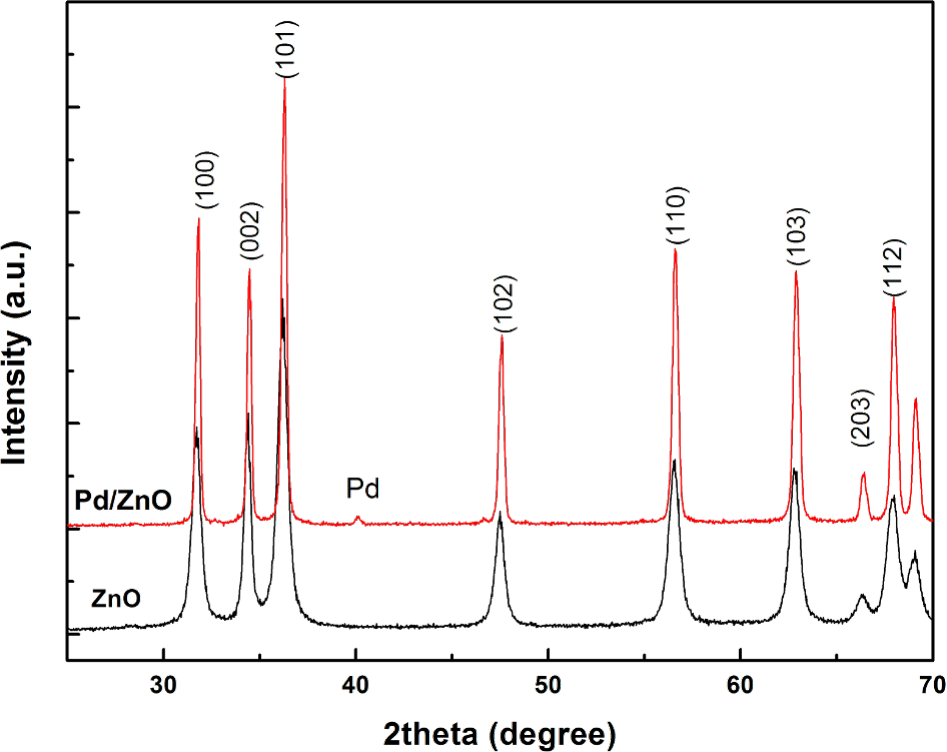
X-ray diffraction patterns of ZnO and Pd/ZnO nanopowders.

X-ray diffraction patterns of ZnO and Pd/ZnO nanoparticles are presented in Figure 1. All the XRD peaks are indexed by a hexagonal wurtzite phase of ZnO (JCPDS card no. 36-1451). The results show that the Pd-doped ZnO sample has a better crystallinity, higher intensity and smaller peak width than those of the pure ZnO sample at 700 °C for 2 hours. For the Pd/ZnO sample, crystalline phases of ZnO and Pd are found to be coexisting. This revealed that metallic Pd nanoparticles are dispersed in the ZnO matrix. The crystallite sizes estimated for the same samples from Scherrer’s formula by using the full width at half maximum (FWHM) [16] from the XRD patterns is in the range of 12 nm to 20 nm, giving average sizes of ZnO and Pd/ZnO samples of 16.2 and 16.5 nm from all the peaks, respectively.

**Figure 2:**
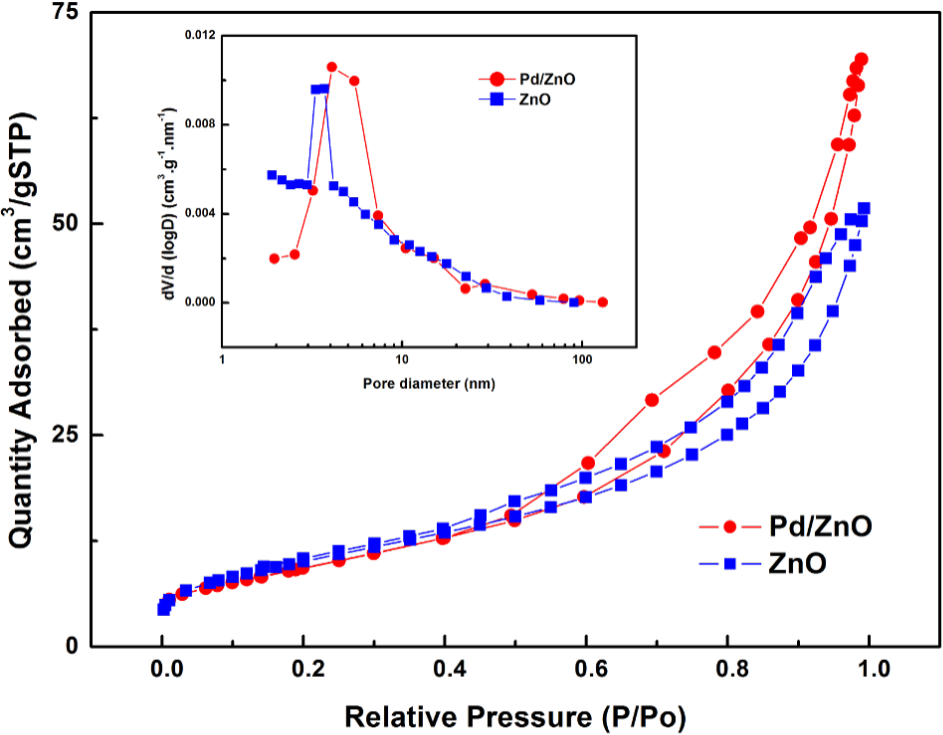
Typical nitrogen adsorption–desorption isotherm and BJH pore size distribution plots (inset) of ZnO and Pd/ZnO nanoparticles.

A more precise determination of the primary particle size is inevitably be accompanied by a significant error due to their aggregate nature and the formation of polycrystalline nanoparticles. Consequently, other methods were used to evaluate the particle size. This includes the Brunauer–Emmett–Teller (BET) surface area analysis and the Barrett–Joyner–Halenda (BJH) pore size and volume analysis. The obtained isotherms of the ZnO and Pd/ZnO samples prepared in ethanol (Figure 2) correspond to a type III isotherm in the Brunauer classification [17–18], which is characterized by the hysteresis loop, and it does not exhibit any limiting adsorption at high relative pressures. The specific BET surface area of the ZnO and Pd/ZnO samples were determined to be 37.5 and 34.32 m^2^/g, respectively, the calculated BJH pore sizes were 8.7 and 10.6 nm. Based on the measured BET surface area the size of the ZnO and Pd/ZnO samples was estimated to be 28 nm and 31 nm, respectively. Thus, BET data satisfactorily correlate with XRD results, and the discrepancy between BET and XRD data can be explained by the complicated geometry of the polycrystalline nanoparticles mentioned above.

**Figure 3:**
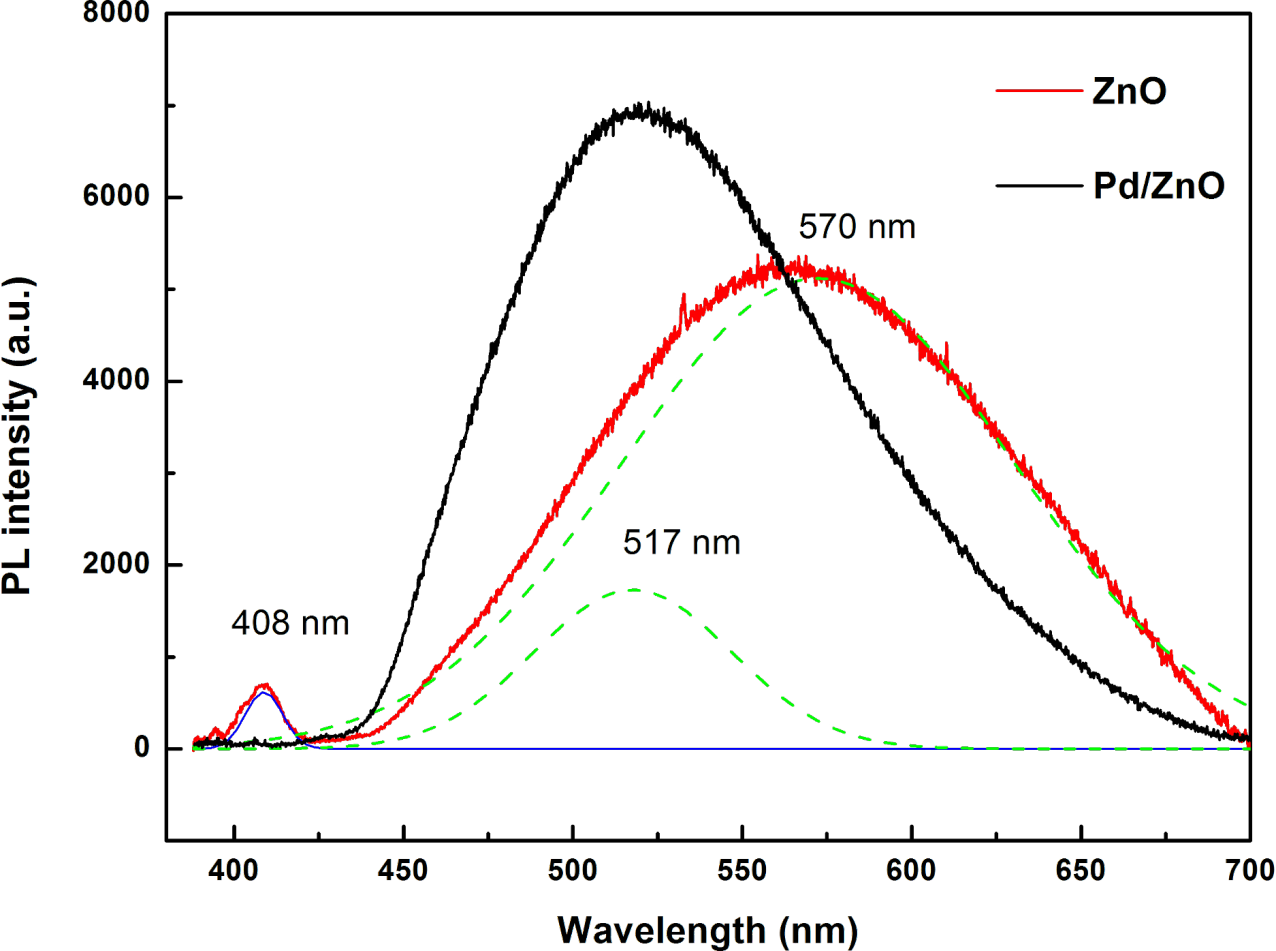
PL spectra of ZnO and Pd/ZnO nanoparticles at room temperature.

To explore the effect of Pd on the optical properties of ZnO nanoparticles, photoluminescence (PL) spectra were measured. Figure 3 shows the PL spectra of ZnO and Pd/ZnO samples with a 325 nm excitation at room temperature. For the ZnO sample a Gaussian fitting analysis shows that the broad emission band is a superimposition of three major peaks, one broad emission with a peak at around 408 nm, a second emission band at 517 nm, and a third emission band at around 570 nm. The UV emission peak at about 408 nm (3.03 eV) corresponds to the near-band-edge emission of the ZnO crystal. The origins of this visible emission have been the subject of a long-standing controversy. It has been attributed to the transition between the electron near the conduction band and the deeply trapped hole, which is an oxygen/zinc vacancy containing no electrons [19–20]. It is also attributed to the transition between donor–acceptor pairs.

In the Pd/ZnO sample, the excitonic band-edge emission completely vanished or was indistinct, while the structured green luminescence band and the transition shift of the emission maximum to higher energies was clearly visible. The second peak at 517 nm increased and the third peak at around 570 nm decreased. Interestingly, the obtained result is confirmed by the similarity of the luminescence bands of ZnO and ZnO:Cu [19–21]. The fine structure is assigned to the longitudinal optical phonon replica with an energy spacing of about 72 meV. This suggests that a green luminescence band originates from palladium ions, which replace zinc and always occur in ZnO in a small amount. The dominant peak at 517 nm corresponds to the exciton transition from the ground-state electronic subband to the ground-state of Pd in replacing Zn sites (i.e., Pd_Zn_ vacancies). The excited state of Pd_Zn_ originates from a hole bound to 4d^10^ shells or an intermediately bound exciton to a neutral d^9^ configuration due to the hybridization of the Pd4d states with the Zn4s states at the bottom of the conduction band. The electron capture takes place at the neutral Pd_Zn_ center (i.e., [Pd^2+^(4d^9^)]), and the hole is captured by the potential created by the tenth electron to form the [Pd^+^(4d^9^+*e*), *h*] state.

**Figure 4:**
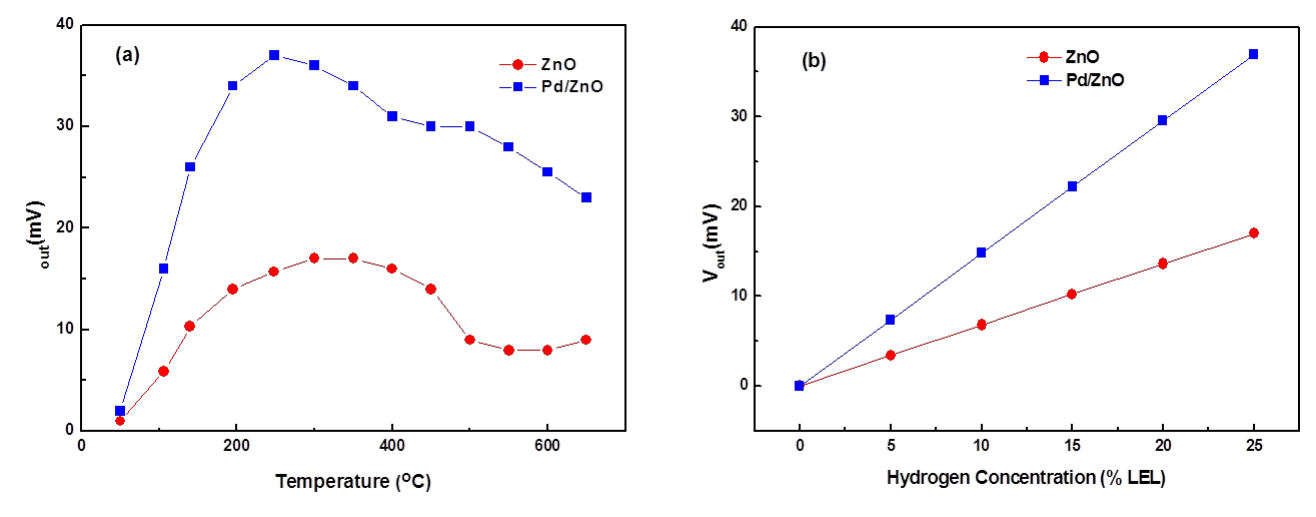
Dependence of (a) the operating temperature on the sensor sensitivity and (b) the hydrogen concentration on the sensor sensitivity (b).

We now discuss the hydrogen sensing characteristics of catalytic gas sensors based on ZnO nanoparticles. For convenience, the fabricated sensors are denoted according to the used sensitive material. that is, sensor 1, ZnO and sensor 2, Pd/ZnO–0.5 wt % Pd. The characteristics of the sensor were examined in a measurement chamber containing hydrogen in the air at 25% of the lower explosive limit concentration (LEL) with a flow speed of 100 mL/min, at a temperature of 30 °C, and a relative humidity of 65% RH. To keep the operating temperature of the sensor in the range of 200–300 °C, an applied voltage *V*_applied_ of 1.7 V and a current of about 120 mA are required. The obtained linear curve implied that it is possible to control the operating temperature by turning the applied voltage. To study the role of the Pd catalyst in the ZnO nanoparticles on the sensitivity of the sensor, the sensor output signal was measured as a function of the operating temperature of the sensor in 25% of the LEL concentration of hydrogen. The temperature of the microheater is tuned by changing the voltage *V*_applied_. The temperature dependence of the sensor sensitivity (through voltage *V*_out_) is shown in Figure 4a.

These results indicate that the sensors have the greatest sensitivity within an operating temperature range of 200–300 °C. Among the measured sensors, the highest sensitivity was found with the Pd/ZnO sample. The values of the output voltage maximum, *V*_out_, were 17 and 37 mV for ZnO and Pd/ZnO samples, respectively. An operating temperature of 250 °C was selected to investigate the gas-sensitive characteristics of the Pd/ZnO sensor. With a flow speed of 100 mL/min and a relative humidity of 65% RH, the linear dependence of the sensitivity of the Pd/ZnO-based sensor on hydrogen concentrations in the range of 0–25% LEL was observed as shown in Figure 4b.

The gas sensing mechanism usually accepted for semiconductor sensors explains the functionality due to reactions of hydrogen with the adsorbed oxygen species (i.e., O^2−^ or O^−^) on the surface of the oxide, which liberate free electrons and H_2_O thereby changing the conductivity of the material. The sensing mechanism for H_2_ at 250 °C can be explained by the Pd metal particles on the surface of ZnO, which act as a catalyst. They dissociate hydrogen molecules into highly reactive atoms, which spread out on the surface of the semiconductor ZnO particles and reduce the potential barrier between the particles. In addition, the greater sensitivity to hydrogen can be explained because the oxidation of dissociated hydrogen is faster and more efficient than the decomposition and oxidation of hydrocarbons [22]. Moreover, as shown in Figure 3, the losing near-band-edge emission and transition shift of green luminescence band are due to Pd_Zn_-vacancies. This allows us to note the correlation between the deep-level emission and the gas-sensing response characteristics of these samples. We suggest that the dissociation of hydrogen takes place at Pd_Zn_-vacancies (i.e, [Pd^2+^(4d^9^)]) and may be expressed as,

[1]
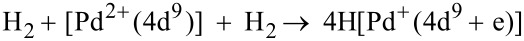


Then, the oxidation of dissociated hydrogen happens according to the reaction,

[2]
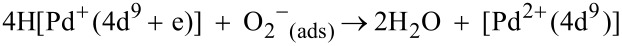


Similar results, viz. that the concentration of vacancies in turn controls the gas sensing characteristics, have been reported in ZnO films [23]. The sensitivity and selectivity characteristics of the gas sensor are associated with the deep hole-trap states and vacancies on the ZnO surface by the electron transfer mechanism [23–24]. The Pd metal nanoparticles modify the charge density on the ZnO surface, so that the incident electric field is changed [25], which not only affects the emission but also changes the oxygen adsorption and desorption of the gas sensors based on Pd/ZnO nanocrystals.

**Figure 5:**
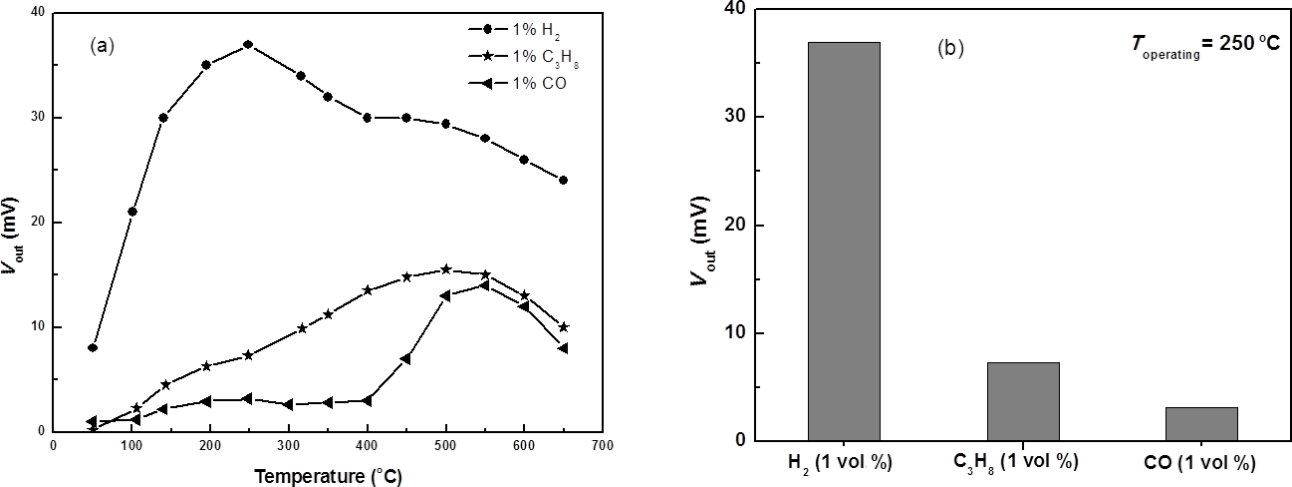
The operating temperature-dependence of (a) the sensitivity and (b) the specificity of the Pd/ZnO sample at 1 vol % of H_2_, CO and C_3_H_8_ with a humidity of 65% RH.

For gas selectivity of the sensor based on Pd/ZnO nanoparticles, the sensitivity of the ZnO– 0.5 wt % Pd sensor depends on the operating temperature. This is shown in Figure 5 for 1 vol % of hydrogen, H_2_, carbon monoxide, CO, and for propane, C_3_H_8_. The comparison of the specificity of the sensor for the studied gases at 250 °C shows that the sensor is highly sensitive to H_2_ and less sensitive to CO and C_3_H_8_. Gas specificity of the sensor is evaluated in terms of the ratio of the sensitivity of the sensor for a particular gas and the sensitivity for other gases when compared under identical conditions (*K*_gas/different gas_). At 250 °C, we found the ratios of *K*_H2/CO_ = 11.5, and *K*_H2/C3H8_ = 5.1. This confirms that the produced sensors display a good specificity for H_2_ compared to the cases of CO and C_3_H_8_. In fact, the effects of these gases on the hydrogen specificity of the sensor is insignificant because the concentration of CO in air is usually very low (<100 ppm), much smaller than a concentration value of 1 vol % (ca. 10,000 ppm).

**Figure 6:**
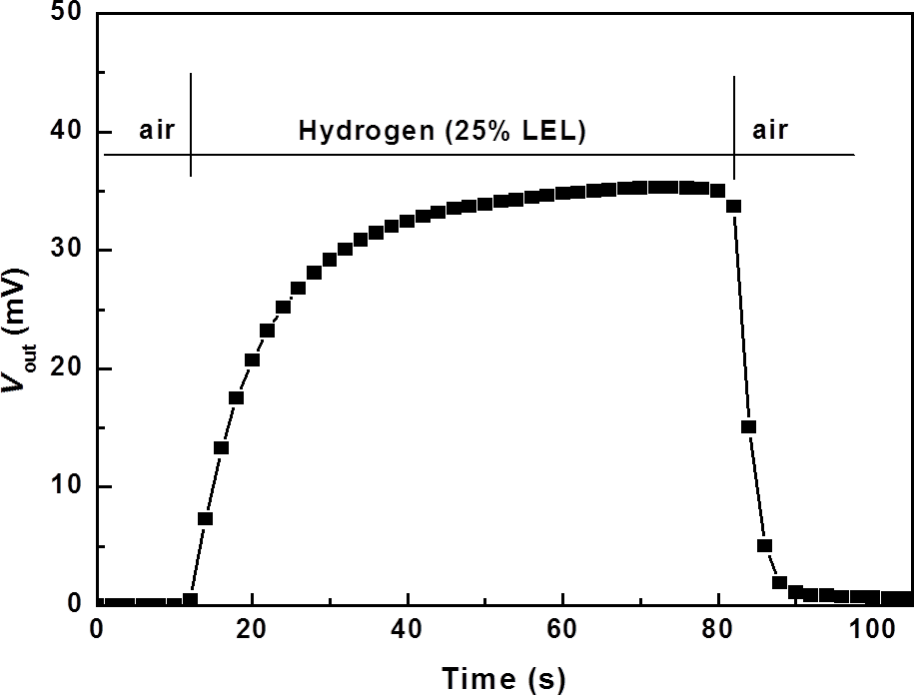
Response characteristics of the hydrogen sensor based on Pd/ZnO nanoparticles.

Figure 6 shows the characteristic of the sensor response to the H_2_ concentration of 25% LEL at 250 °C. The results show that the response time of the sensor is in the range of 10–20 s and the recovery time is around 10 s. Thus, this sensor is quite suitable for designing portable equipment as well as online control equipment to measure H_2_ concentrations in the range of 0–100% LEL.

**Figure 7:**
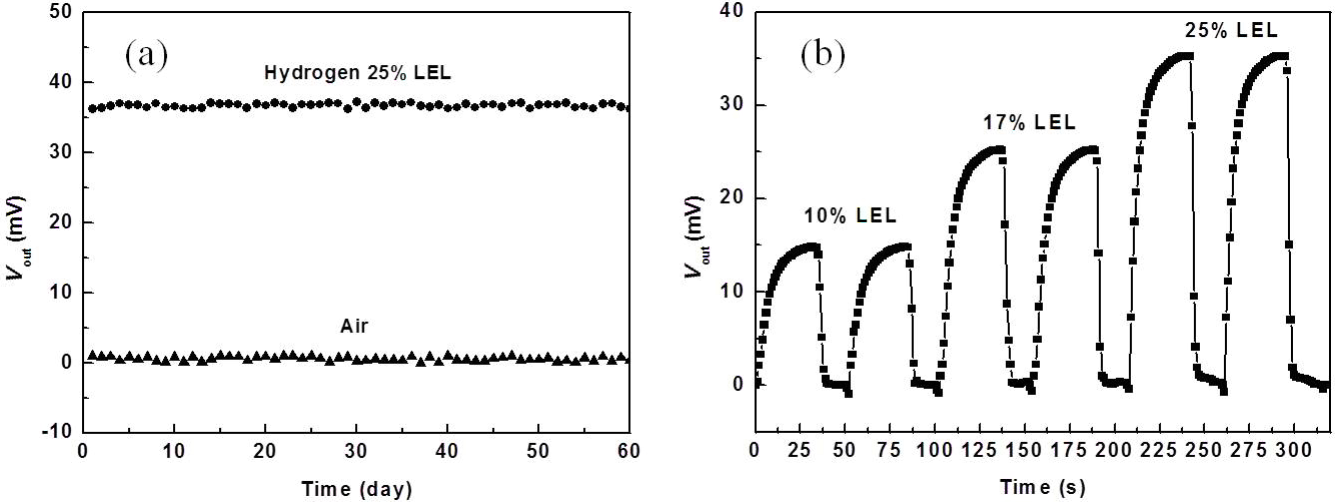
Stability over time of the investigated sensor as a function of (a) time and (b) in hydrogen atmosphere.

Finally, to examine the stability of the sensor over time, the measured value of the sensor is recorded every day under two atmospheric conditions, namely, in air and in H_2_ concentrations of 25% LEL. The monitoring was carried out for 60 days, the results show that the sensor is rather stable with measured values fluctuated in the range of ±3 mV (Figure 7a). The stability of the sensor was also investigated by measuring a cycle at different concentrations of H_2_ (as shown in Figure 7b). For each concentration of H_2_, measurements were performed twice with an interval time of 20 minutes to minimize the effect of the previous measurements. The results show that the measured values are stable.

## Conclusion

ZnO and Pd/ZnO (with 0.5 wt % Pd) nanoparticles with a nanoscale particle size of 16.2 and 16.5 nm and with a large specific surface area of 37.5 and 34.32 m^2^/g, respectively, were prepared by wet chemical methods for gas sensor fabrication. The PL spectra at room temperature show that the carrier dynamics coincides with the buildup of the Pd-related green emission. A novel gas sensor based on 0.5 wt % Pd mixed with ZnO nanoparticles exhibited a high response to hydrogen at a relatively low temperature, from 200 to 300 °C, and the best operating temperature of the sensor is at 250 °C. We also showed that a qualitative correlation exists between the deep level emission and the gas sensing response characteristics of these samples. We suggest that the dissociation of hydrogen takes place at Pd_Zn_-vacancies ([Pd^2+^(4d^9^)]). The design of sensors as a catalytic membrane and Wheatstone bridge measurements allow for the continuous monitoring of the H_2_ concentration in the range of 0–100% LEL with high sensitivity and selectivity.

## Experimental

ZnO nanopowders were synthesized by a wet chemical method with zinc acetate dihydrate, Zn(CH_3_COO)_2_·2H_2_O, sodium hydroxide, NaOH, and absolute ethanol (analytical reagents, Merck) as starting materials. In a typical procedure, 1.314 g of zinc acetate was dissolved in 300 mL solvent in a three-necked flask under stirring at 60 °C. 0.48 g of sodium hydroxide was added into this solution. After stirring for several minutes, a white precipitate appeared. The solution was stirred at 60 °C for 1 hour. After washing several times with distilled water and absolute ethanol, the particles were dried at 80 °C for 12 h. Pd-doping samples (Pd/ZnO) were obtained by mixing ZnO nanopowder with palladium chloride (Pd content in the sample is of 0.5 wt %). All samples were calcined at 700 °C for 2 hours to decompose the original chloride to obtain Pd/ZnO. The hydrogen sensors were improved on a pellistor gas sensor [26], which consisted of two resistors with R = 500 Ohm, two platinum coils. Their thick membrane (about 10 μm) of ZnO pastes with/without Pd was coated onto one platinum coil (activated bead). Al_2_O_3_ paste was coated onto the last coil. The temperature of the beads is controlled by the operational electrical current passed through the platinum coils. At high temperatures the chemisorbed hydrogen molecules on the surface catalyst are oxidized with adsorbed oxygen to form water. The heat of combustion raises the temperature of the activated bead, which in turn changes the resistance of the activated coil. This creates an imbalance in the Wheatstone bridge circuit. In this case, the offset voltage is measured as the signal instead of resistance or conductivity values.

The temperature of the sensor was controlled by a UDP-1501 power supply (Unicorn, Korea) connected with a computer, and data were recorded automatically by using a Keithley model DMM-2700.

Crystalline phase analyses of synthesized samples were characterized by powder X-ray diffraction (XRD) with a Siemens D5000 diffractometer by using CuK_α_ radiation. Morphologies of the samples were obtained by a Field Emission Scanning Electron Microscope (FE-SEM), Hitachi S-4800S. The specific surface areas of the prepared samples were determined by the BET (Brunauer–Emmett–Teller) method on a micromeritics system - AutoChem II 2920. The photoluminescence (PL) signals were dispersed by using an 1800 grating monochromator (Horiba iHR550) and then detected by means of a thermoelectrically cooled Si-CCD camera (Synapse).
